# Associations between Race, Discrimination, Community Violence, Traumatic Life Events, and Psychosis-Like Experiences in a Sample of College Students

**DOI:** 10.3390/jcm8101573

**Published:** 2019-10-01

**Authors:** Pamela J. Rakhshan Rouhakhtar, Steven C. Pitts, Jason Schiffman

**Affiliations:** Psychology Department, University of Maryland, Baltimore County, 1000 Hilltop Circle, Baltimore, MD 21250, USA; rakhshp1@umbc.edu (P.J.R.R.); spitts@umbc.edu (S.C.P.)

**Keywords:** psychosis-like experiences, race, measurement validity, principal components analysis, discrimination, trauma, community violence

## Abstract

Self-report tools of psychosis-like experiences contribute to the understanding of psychosis and may aid in identification and prevention efforts across the severity spectrum. Current tools are likely limited by biases, leading to potential systematic health disparities. Principal component analyses in diverse samples of community participants reporting psychosis-like experiences may aid in the detection of measurement biases. The current study evaluated the fit of a two-component model for the Prime Screen, a self-report psychosis-like experiences measure, in a sample of Black (*n* = 82) and White (*n* = 162) community participants, and subsequently evaluated the relation of these components with measures of mental well-being, traumatic life experiences, community violence, and experiences of discrimination. Analyses indicated limited support for a two-component model of the Prime Screen, with four of the items showing high cross-loading across both components (“poor fit” items). Although many Prime Screen items correlated with mental well-being as expected, correlations between item scores and mental well-being were non-significant for poor fit items. Community violence emerged as a significant predictor of some individual item scores for both good and poor fit items, while discrimination predicted only some poor fit item scores. Results highlight the potential limitations of current self-report tools of psychosis-like experiences, as well as possible considerations for improvement for use in diverse populations.

## 1. Introduction

Psychotic disorders, though heterogenous in both clinical presentation and trajectory, share a common definition, as follows: Presence of either delusions and/or hallucinations, with additional symptoms like disorganization in speech/thought/behavior, motor disturbances, and negative symptoms, sometimes comorbid [1]. Early clinical work and theory largely focused on chronic severe forms of psychotic illness like schizophrenia, likely due to the often pervasive, devastating, and long-lasting effects of these disorders on the lives of individuals with psychotic illness [2,3,4,5,6]. Increasing interest has focused on earlier phases of illness as well as manifestations of symptomatology that are less severe than chronic/acute psychosis, in the hopes of both furthering understanding of the nature of psychosis across the severity spectrum, as well as developing and testing preventative efforts for psychotic illness.

In keeping with recent paradigms in theoretical psychopathology [7,8], psychosis is thought to exist on a spectrum, with transient or subclinical symptoms, known as psychosis-like experiences, having higher lifetime prevalence in the population (~20% of children and adolescents [9,10] and ~8% of adults [11,12]) compared to diagnosed psychotic disorders (~3% of adults [13]). Requisite for diagnosis of a psychotic disorder are not just symptoms of psychosis per se, but also clinically significant levels of distress or impairment resulting from these symptoms. Although not frequent or severe enough to meet criteria for a clinical diagnosis, psychosis-like experiences (PEs) that are subclinical manifestations of hallmark psychotic symptoms (e.g., perceptual disturbances vs. hallucinations) may also cause significant distress and impairment. The overwhelming majority (~80%) of PEs are transient in duration and impact and the vast majority of individuals who experience PEs will not develop a psychotic disorder, yet those with PEs are more likely to experience more severe symptoms of psychopathology, functional impairment, and to need psychological intervention compared to peers with no/low levels of PE [14,15,16,17,18].

In addition to the phenomenological similarities, in a systematic review and meta-analysis van Os and colleagues (2009) reported that individuals across the psychosis severity spectrum showed similarities regarding clinical and functional domains [11]. People along the psychosis spectrum also share genetic and non-genetic risk factors. Collectively, this body of research suggests that those with PEs have the potential for common developmental pathways as in those with psychotic disorders. Given the distress and impairment associated with PEs, the more recent understanding of psychosis and other psychopathology as phenomenon existing on a spectrum rather than a categorical construct and the difficulties in participant recruitment, given the low prevalence and high functional impairment often associated with psychotic disorders, research among individuals with PEs is of clinical and conceptual relevance in understanding the broader psychosis spectrum.

### 1.1. Self-Report Measures

Allowing for broader identification efforts than clinician interviews, self-report measures of PEs and attenuated symptoms of psychosis represent a promising first step in specialized early identification models for psychosis. To date, several self-report screening tools for these purposes have been developed [19]. Screening measures, such as the Prodromal Questionnaire-Brief [20] or the Prime Screen [21], generally show good agreement with clinical interviews and adequate levels of sensitivity and specificity [22,23]. Additionally, use of screening tools during sample recruitment seems to result in the identification of more instances of positive risk for the future development of diagnosable psychosis than methods without utilization of screening measures [23,24,25]. The Prime Screen, developed by the authors of the Structured Interview for Psychosis Risk Syndromes (SIPS; [21,26]) offers several advantages, such as its brevity (12-item scale), theoretical similarity to SIPS positive symptom items, and similar psychometric performance compared to other PE and risk screening tools [27]. Since its development, the Prime Screen has been used in a number of diverse samples and research studies [22,27,28,29,30].

Despite the advances made towards the validation of tools like the Prime Screen, additional work is required to refine measure performance across multiple populations. Variability of psychometric performance of the Prime Screen indicates the potential presence of measurement error or moderators of measure effectiveness. Although self-report tools perform well in some samples, PE and psychosis risk screening measures like the Prime Screen are often hampered by high false positive rates [22,27,28]. Cultural or racial biases may represent one factor that contributes to the poor psychometric performance among PE self-report tools, as well as the variability seen among studies in measure predictive validity.

### 1.2. Racial and Cultural Biases in PE/Psychosis Risk Self-Report Tools

Disparities in measurement validity have been demonstrated among race groups for the Prime Screen. Although these may reflect differing rates of psychotic illness in racial minority groups due to risk factors such as discrimination, migration, or elevated exposure to trauma [31], differences may reflect a limited generalizability of the Prime Screen to diverse individuals. Millman and colleagues (2019) [32], examining an adolescent help-seeking sample, found that Prime Screen performance, when predicting clinical interview identified risk for psychosis, differed significantly based on participant race. Although Prime Screen scores showed strong predictive values for clinical interview diagnosis among White participants, screening measure scores were not a significant predictor of risk status for Black participants. Item level analysis showed that mean differences between those at risk and those not at risk varied significantly by race. Whereas White at-risk participants often showed higher mean scores for Prime items as compared to their low-risk peers, Black at-risk participants often displayed similar or even lower mean item scores on the Prime Screen as compared to Black low-risk participants on select Prime Screen items.

Differences in Prime Screen performance among varied racial/cultural groups have been demonstrated in other multinational samples. Although results indicated robust psychometric properties overall, validation of the Prime Screen in a Japanese clinical sample yielded relatively low concordant validity (0.43) with clinician determined risk for psychosis, indicating the potential for false positive identification of individuals within this sample. Cluster analysis of Prime Screen measure scores performed by Mamah and colleagues (2012) [33] in a Kenyan sample identified four differential respondent patterns, as follows: (1) High symptoms, (2) low/intermediate symptoms, (3) high grandiosity, and (4) normative/no symptoms. The presence of response styles, such as low/intermediate symptoms or high grandiosity, indicate the potential for the Prime Screen items to reflect phenomena other than symptoms of risk or PEs that are clinically meaningful—That is, it is unclear whether these respondents showed patterns reflective of underlying dimensional psychotic presentations or if the differences between high and no symptom participants indicate the measurement of other constructs, such as cultural beliefs. In another study of the same sample, Owoso and colleagues (2014) [29] evaluated the psychometric performance of the Prime Screen, finding lower levels of sensitivity (40%), specificity (64%), PPV (Positive Predictive Value; 12%), and NPV (Negative Predictive Value; 90%) when predicting interview determined risk for psychosis status as compared to previous work. Additionally, a number of correlations between items on the Prime Screen and clinical interview items were nonsignificant, specifically those probing superstitions, wondering whether experiences were real or imaginary, mind reading, presence of special or supernatural gifts, or hearing one’s thoughts out loud. These findings seem to indicate the potential for embedded cultural biases to impact Prime Screen psychometric validity when used across diverse groups. 

Recent work by Rakhshan Rouhakhtar and colleagues (in preparation) [34] may highlight what could be one mechanism behind measurement biases seen in the Prime Screen for Black individuals as compared to their White counterparts. In two independent and racially diverse help-seeking samples (*n* = 97; *n* = 499), a principal components analysis and subsequent confirmatory factor analysis were estimated to identify the underlying structure of the Prime Screen, with further criterion validity analyses performed. A two-component structure of the Prime Screen emerged. For one of the components (“psychosis-risk symptoms,” items 1, 3, 5, 7, 9, 10, 11, and 12), higher scores significantly predicted early psychosis/risk and social functioning deficits in one sample and global mental health, well-being, psychiatric symptom, and life functioning deficits in the other, suggesting good validity. For the other component (“poor fit items,” items 2, 4, 6, and 8), which included questions regarding participants’ experiences regarding mind reading, superstitions, predicting the future, and possession of supernatural gifts, no significant relations were observed with early psychosis/risk and no significant associations emerged between mental health or functioning scores and component 2 scores. These results highlight the potential limitations of the Prime Screen and may suggest that some items currently included in the measure may have a relative lack of validity; particularly for diverse populations. The limited ability to draw conclusions regarding why component 2 items performed poorly, and to test associations between component 2 scores and potential confounds, including cultural or race-related factors, indicates that future work should expand upon these initial findings to address these questions.

### 1.3. Racial Biases in Attenuated Psychosis Screening Tools: Trauma

Psychosis and trauma are often linked, reflective of both the broader connection between trauma and psychopathology, as well as a unique, potentially mechanistic, link between traumatic life events and psychosis [35]. Although trauma impacts individuals across all race groups, racial disparities exist regarding the prevalence or implications of traumatic experiences. In a large study of trauma exposure in the United States, African-Americans were more likely to have a lifetime prevalence of post-traumatic stress disorder (PTSD; 8.7%) as compared to their other race peers (between 4%–7.4% among different race groups). Additionally, African-American individuals had a higher likelihood of exposure to child maltreatment, compared to their White peers, and were less likely to seek treatment for PTSD than their White peers [35]. Although a number of factors are related to this differential pattern, discrimination may play a key role. In a longitudinal study of African-American and Latinx individuals, discrimination significantly predicted PTSD diagnoses but not anxiety or mood disorders, indicating that discrimination may be uniquely related to trauma-related disorders [36]. Responding to this evidence, a growing body of work has begun to examine the relation between discrimination and trauma, with some arguing that consistent instances of racial discrimination and harassment may be conceptualized as traumatic experiences that may lead to trauma-related disorders like PTSD, or symptoms similar to these illnesses [37,38,39,40,41,42].

Given the racial disparities seen in trauma experiences and diagnosis rates, the unique risk factors associated with racial minority status for trauma-related disorders, and the growing consideration among scholars of racial discrimination as a trauma per se, it is important for the development and use of attenuated psychosis self-report measures to understand the unique associations between responses on these scales and race, trauma, and discrimination.

### 1.4. Current Study

In response to the relative lack of research evaluating the factor structure of the Prime Screen and other PE and psychosis-risk self-report measures, as well as the significant need to improve the psychometric properties of these self-report tools through evaluation of the impact of relevant factors such as discrimination, trauma, and race on measurement validity, the current study attempts to address this gap by (1) evaluating the fit of a 2-component structure of the Prime Screen and (2) examining the associations between item scores across race groups with measures of mental well-being, trauma, community violence, and racial discrimination. This work will aid in identifying whether item scores on this commonly used self-report tool are related to self-reported measures of mental well-being. Additionally, if item content is reflective of factors other than psychosis symptomatology, including trauma and discrimination, these findings may have implications for the use of current screening measures like the Prime Screen in diverse populations and could highlight the need for further work or the improvement of self-report tools.

## 2. Method

### 2.1. Participants

This study was conducted at the University of Maryland, Baltimore County (UMBC), with procedures reviewed and approved by the UMBC institutional review board. AN Inclusion criterion for participation was an age of 18 years or greater, with further criteria for inclusion in the analysis sample including self-reported race of White or Black and completion of the Prime Screen. Participants were not excluded from the current study for current mental health treatment. Participants (*N*_consented_ = 478, *N_analysis_* = 244) were recruited from an online UMBC student volunteer pool.

### 2.2. Measures

Inclusion criteria screening, consent, and study participation all occurred online through a Qualtrics survey platform. After initial consent, participants were given a period of 7 days to complete the study before the survey record was closed. Participation was reimbursed with course credit. Participants were required to submit responses to all questions to complete the study, with each question including a “prefer not to respond” option.

#### 2.2.1. Demographics

Measures of race, gender, birth date, mental health treatment history, and family mental-health history were self-reported by participants. Race was defined as “Black or African American” and “White or Caucasian”.

#### 2.2.2. Prime Screen

The Prime Screen is a self-report measure of presence and severity of psychosis-risk symptoms/PEs (Miller et al., 2004) [21]. The measure contains 12 Likert-type items, with response options ranging from 0 (“definitely disagree”) to 6 (“definitely agree”), and has demonstrated adequate psychometric performance relative to clinician interview diagnoses of risk (Kline et al., 2012) [27]. For the current study, both the total score of the Prime Screen and dichotomous at-risk status (those with ≥1 response at a level of 6 (“definitely agree”) or ≥2 responses at a level of 5 (“somewhat agree”) were considered to be “at-risk”) were calculated.

#### 2.2.3. Life Events Checklist (LEC)

The Life Events Checklist (LEC) is a self-report measure of potentially traumatic events containing 16 items in which participants indicate whether each event happened to them, was witnessed, or the participant learned about it happening to someone close to them during their lifetime [43]. The LEC has demonstrated psychometric validity, with responses strongly related to both psychological distress as well as PTSD symptoms [43]. The present study created a total score by summing the total number of lifetime events experienced across self-response options.

#### 2.2.4. Experiences of Discrimination (EOD)

Discrimination was measured using the Experiences of Discrimination (EOD) questionnaire, a self-report measure of a number of constructs relating to discrimination, including experiences of situational discrimination, frequency of discriminatory experiences, response to discrimination, and worries about discrimination [44]. The present study only included the 9-item situation section, summing the total number of situations in which participants positively endorsed experiences of discrimination due to their race. Previous work has found evidence for the valid and reliable use of the EOD questionnaire as a self-report measure of discrimination [44].

#### 2.2.5. Community Experiences Questionnaire (CEQ)

The Community Experiences Questionnaire (CEQ) is a 25-item self-report measure of individuals experiences of community violence, with two subscales on the frequency with which individuals were either directly victimized by or witnessed community violence ranging in severity from threats to killings [45,46]. Participants respond to each item on a 4-point Likert-type scale, with 1 indicating they “never” had this experience and a 4 indicating it had occurred “lots of times”. Participants were asked to rate their lifetime exposure to events listed in the questionnaire. For the current analyses, a total community violence score was calculated by averaging answers across all items. The CEQ was developed by Schwartz and Proctor (2000), who adapted and simplified the language of validated community violence measures for easier use in urban multiethnic populations. 

#### 2.2.6. Mental Health Continuum-Short Form (MHC-SF)

The Mental Health Continuum-Short Form (MHC-SF) is a 14-item 6-point Likert-type scale measuring well-being and positive mental health, with sub-dimensions of the scale including emotional well-being, psychological well-being, and social well-being [47]. Participants indicate the frequency of each item over the past month, with response options ranging from 0 (“never”) to 5 (“every day”). For study analyses, mental well-being was defined as the total score of all MHC-SF items. Research has indicated the MHC-SF has adequate psychometric properties and may be considered a reliable measure of mental well-being [47].

### 2.3. Statistical Analyses

All analyses were performed using SPSS version 25. Primary study variables were visually and numerically examined for normality and outliers, with any significantly non-normal variables transformed before analyses as applicable. Group differences in demographic and primary study variables by race were evaluated. When applicable, we evaluated homogeneity of variance prior to tests of mean difference using Levene’s test, with the assumption of homoscedasticity considered to be violated for all tests with *p* < 0.05. In the event of heteroscedasticity, a Welch–Satterthwaite adjustment to the degrees of freedom was used. 

All primary analyses were estimated separately for Black and White participants. Principle components analyses (PCA) of the 12 Prime Screen items were estimated in the Black and White samples. A Kaiser–Meyer–Olkin (KMO) and Bartlett’s Test of sphericity were calculated to evaluate sample size adequacy and sphericity. The 12-item correlation matrix was used as the association matrix. Given the exploratory nature of the current study and the lack of established factor structure for the Prime Screen, principle components analysis with quartimax rotation was used to extract the components, with a fixed number of two components specified based on previous component analysis of the Prime Screen [34]. Item component loadings were evaluated for simple structure, with item loadings of ≥ 0.5 on a single component considered meaningful; items with component loadings ≥ 0.32 on more than one component were deemed cross-loaded items [48].

To evaluate the relation between item scores and other relevant constructs, correlations were estimated between normalized Prime Screen items and mental well-being scores. Four multivariate linear regression models were estimated. One model was estimated for both Black and White participants separately, with total lifetime traumatic experiences, discrimination total score, and community exposure to violence mean score predicting Prime Screen item scores with strong component loadings (Prime Items 1, 3, 4, 5, 9, 10, 11, and 12). A second model was estimated for both Black and White participants separately, with total lifetime traumatic experiences, discrimination total score, and community exposure to violence mean scores predicting and the Prime Screen item scores with high cross-loadings or poor fit (Prime Items 2, 6, 7, 8).

## 3. Results

### 3.1. Preliminary Analyses

Tests of the relation of race with: gender (*χ*^2^(1, *n* = 243) = 0.02, *p* = 0.88), family history of schizophrenia (*χ*^2^(1, *n* = 229) = 0.78, *p* = 0.38), Prime Screen risk status (*χ*^2^(1, *n* = 244) = 1.28, *p* = 0.26), Prime Screen total score (*t*(242) = 0.15, *p* = 0.88), mental well-being total score (*t*(229) = −0.32, *p* = 0.75), community exposure to violence scores for victimization (*t*(226) = −0.79, *p* = 0.43), and total sum of traumatic experiences—Self (*t*(242) = −1.23, *p* = 0.22) suggested no race differences for these variables.

Significant race differences emerged, however, for age (*t*(240.72) = −2.35, *p* = 0.02), mental health treatment history (*χ*^2^(1, *n* = 229) = 6.72, *p* = 0.01), community exposure to violence scores for witnessed (*t*(226) = −2.00, *p* < 0.05), and experience of discrimination scores (*t*(92.28) = 5.10, *p* < 0.001), with Black participants showing younger age (*M*_Black_ = 21.11, *M*_White_ = 22.44), lower rates of mental health treatment in the past 2 months (%_Black_ = 13.33, %_White_ = 28.85), lower mean scores for exposure to community violence—witnessed (*M*_Black_ = 1.47, *M*_White_ = 1.62), and higher total discrimination scores (*M*_Black_ = 1.81, *M*_White_ = 0.54). For descriptive statistics and frequencies for the analysis sample, see Table 1.

### 3.2. Principal Components Analysis

Prior to analyses, inter-item correlations, descriptive statistics, and normality estimates were generated for all Prime Screen items (see Table 2). All scale items were highly correlated. For the 12-item scale, Chronbach’s alpha was 0.87 in the total sample, with reliability statistics similar across race groups (α_Black_ = 0.87, α_White_ = 0.86). Given the non-normal distribution of all Prime Screen items in the analysis sample, all item data was transformed (natural log transformation of Prime Item score + constant [1]) before principal components analysis was performed. The acceptability of the current sample for PCA was confirmed with a KFO of 0.88 and a Bartlett’s test of sphericity yielding χ^2^ (66, *N* = 82) = 399.59, *p* < 0.001 for Black participants, and similarly with the White sample, KFO = 0.89 and χ^2^ (66, *N* = 162) = 739.72, *p* < 0.001.

Principal components analysis with quartimax rotation and a fixed number of components (2) yielded a solution that accounted for 54.17% of the total Prime Screen variance in the Black sample and 53.86% of the score variance in the White sample (see Table 3 for component loadings).

Across both race groups, items 1, 3, 4, 5, 9, 10, 11, and 12 demonstrated simple structure with respect to component 1. In the Black participant group, item 7 showed high levels of cross-loading across components and items 2, 6, and 8 showed high cross-loading within both race groups, indicating poor item fit for the two-component model. Exploratory analyses of a one-component solution indicated that this model did not fit the data better than the two-component solution, with a number of items showing low communality values (< 0.40) in both the Black participant model estimate (Items_Communality < 0.4_ = 5) and the White participant model (Items_Communality < 0.4_ = 3).

### 3.3. Item Scores and Mental Well-Being

Correlations between transformed item scores and mental well-being were estimated (see Table 4).

For participants who self-identified as Black, only item Prime item 12 (“I have been concerned that I might be ‘going crazy’”) was significantly correlated with mental well-being scores (*r* = −0.23, *p* < 0.05). For White participants, scores for item 1 (“I think that I have felt that there are odd or unusual things going on that I can’t explain”; *r* = −0.22), item 3 (“I may have felt that there could possibly be something interrupting or controlling my thoughts, feelings, or actions”; *r* = −0.19), item 4 (“I have had the experience of doing something differently because of my superstitions”; *r* = −0.17), item 5 (“I think that I may get confused at times whether something I experience or perceive may be real or may just be part of my imagination or dreams”; *r* = −0.22), item 7 (“I wonder if people may be planning to hurt me or even may be about to hurt me”; *r* = −0.24), item 9 (“I think I might feel like my mind is ‘playing tricks’ on me”; *r* = −0.33), and item 12 (“I have been concerned that I might be ‘going crazy’;” *r* = −0.32) were significantly correlated with mental well-being scores, all *p*s < 0.05.

### 3.4. Item Scores and Trauma, Community Violence, and Discrimination

Multivariate linear regression models were estimated to determine the relation between Prime Screen item scores and trauma, community violence, and discrimination. Separate models in which traumatic life events, experiences of discrimination, and community violence were predictors of transformed item scores for items with good fit (evidence of simple structure; items 1, 3, 4, 5, 9, 10, 11, and 12; see Table 5) and items with poor fit (items 2, 6, 7, and 8; see Table 6) were estimated for each race group.

For good-fit item models, no statistically significant predictors emerged in the Black participant group (all *p*s > 0.05), while community violence significantly predicted item scores for White participants (*F*(8, 134) = 5.06, *p* < 0.001, *η_p_*^2^ = 0.23).

For poor fit items models, discrimination was a significant predictor of item scores for Black participants (*F*(4, 61) = 4.81, *p* < 0.01 *η_p_*^2^ = 0.24), whereas discrimination (*F*(4, 138) = 5.95, *p* < 0.001, *η_p_*^2^ = 0.15) and community violence (*F*(4, 138) = 3.27, *p* < 0.05, *η_p_*^2^ = 0.09) were significant predictors of item scores for White participants.

## 4. Discussion

Given the difficulty faced by clinical scientists in distinguishing between pathological and cultural and/or contextual experiences within the spectrum of psychosis during measurement construction and validation [49], data-driven methodologies may be used as a complement to traditional psychometric studies to evaluate the construct validity of screening measures of PEs and risk for psychosis and their items. Analytical techniques like principal components analysis can aid in the identification of measurement bias in early psychosis measures [50,51,52]. In the current study, we used a principal components analysis to evaluate the fit of items on the Prime Screen onto a two-component solution in a sample of Black and White college students. We subsequently evaluated the relation between item scores and measures of mental well-being, traumatic life events, discrimination, and community violence exposure. We found limited evidence for a two-factor model of the Prime Screen in the current sample of Black and White participants, with subsequent analyses revealing that Prime Screen items were differentially related to mental well-being, community violence, and discrimination. Additionally, the strength and/or statistical significance of the correlational analyses for mental well-being and Prime Screen items differed across race groups, seeming to indicate there may be psychometric differences for the Prime Screen when used with Black vs. White individuals. The present findings are in keeping with previous work demonstrating validity concerns that may be related to cultural or contextual factors at the factor level for the Prime Screen or other PLE/attenuated psychosis self-report measures [34,50,51].

### 4.1. Component Solution and Item Fit

Findings suggest that, in the current sample, there is limited evidence of the acceptability across race groups for a two-component solution for the Prime Screen. Items 2 (“I think that I might be able to predict the future”), 6 (“I have thought that it might be possible that other people can read my mind, or that I can read other’s minds”), and 8 (“I believe that I have special natural or supernatural gifts beyond my talents and natural strengths”) showed poor fit within both race groups. Item 7 (“I wonder if people may be planning to hurt me or even may be about to hurt me”) displayed simple structure in the White participant sample, but a cross-loading in the Black participant group. Of note, exploratory analyses of a one-component structure across race groups found low communality values for a number of items in both Black (*n*_items_ = 5 with communality < 0.4) and White participants (*n*_items_ = 3 with communality < 0.4), seemingly indicating that a one component structure did not fit the data better than the two-component solution.

Previous work by our team in two racially diverse help-seeking samples (Rakhshan Rouhakhtar et al., in preparation [21]) found evidence for a two-component solution for the Prime Screen. Notably, the component consisting of items 2 (future prediction), 4 (superstitions), 6 (mind reading), and 8 (supernatural gifts) showed no relation with psychosis or clinical-high risk diagnoses, functioning scores, or self-reported mental health scores. Moreover, component scores comprised of the remaining Prime Screen items showed significant associations with psychosis and psychosis-risk diagnoses, functioning, and mental health scores in the expected direction (endorsement of more severe item scores related to greater likelihood of diagnoses or worse functioning/mental-health scores). This preliminary work, as well as other findings of measurement variance among different race groups for other measures of psychosis experiences [50,51,53], seems to indicate that more work is needed to ensure measures of psychosis are valid and accurate for diverse groups across the help-seeking spectrum.

### 4.2. Prime Screen Items and Mental Well-Being

In the current study, evaluation of Prime Screen item correlations with mental well-being scores indicated that across both race groups, items 2, 6, 8, 10, and 11 were not significantly related to self-reported mental well-being. It is important to note, however, that correlations were of approximately small effect size (*r* = 0.1) or greater for item 10 (hearing talking/mumbling) and item 11 (own thoughts said aloud) in both race groups, and item 8 (special/supernatural gifts) in White participants only, indicating that significant correlations may not have been detected in the current study due to the limited sample size.

Items 2 (future prediction) and 6 (mind reading), however, showed non-significant correlations with mental well-being scores with effect sizes well below that of a small effect for both race groups (between *r* = −0.01 to *r* = −0.04), with items 4 (superstitions; *r* = 0.06), 7 (hurt/harm; *r* = −0.08), and 8 (supernatural gifts; *r* = 0.01) showing less than small effect sizes in Black participants. Results seem to suggest that items 2 and 6 may be candidates for elimination or revision for the Prime Screen pending additional similar findings. Items 4, 7, and 8 may also fall into this category on the basis of limited associations in the current sample with mental well-being among Black participants.

Examining differences in item association across race groups further, there were differential patterns in the magnitude of the relation between mental well-being scores and Prime Screen items in the Black and White participant groups, with differences of ≥0.1 between correlations across race groups for item 4 (superstitions), item 7 (others planning to hurt), and item 9 (mind tricks), with increases in Prime Screen item scores more strongly associated with mental well-being deficits in White participants as compared to their Black peers. These findings are in keeping with other work demonstrating racial differences in the Prime Screen [32] and, taken with the current findings of validity concerns across race groups for some Prime Screen items, support the possibility that measurement biases may exist in this measure of psychosis experiences both as a function of racial factors, as well as other sources present across race groups, including trauma, discrimination, and community violence.

### 4.3. Traumatic Life Events, Discrimination, and Community Violence

Multivariate multiple linear regression models predicting items that displayed simple structure and “poor fitting” items within both race groups indicated that for items that demonstrated simple structure (items 1, 3, 5, 9, 10, 11, and 12), only community violence was a significant predictor of item scores for White participants and none of the measures (traumatic life events, experiences of discrimination, nor community violence) were significant predictors of individual items for Black participants. For items with poor fit onto the two-component solution (items 2, 6, 7, and 8), however, experiences of discrimination emerged as significant predictors of item scores for both Black and White participants, while community violence was a significant predictor for poor fitting items as well for White participants. These findings suggest that self-reported exposure to community violence is related to Prime Screen item scores across good and poor fit items for White participants, while experiences of discrimination are related to Prime Screen item scores across both race groups for those items we found to fit poorly onto the two-component solution in the current analysis. This is in keeping with the broader literature on psychosis experiences, discrimination, and community violence, given that a number of studies have linked both experiences of discrimination [54,55,56], as well as community violence [57,58,59], with the endorsement of psychosis experiences.

In all models, when traumatic life events, experiences of discrimination, and community exposure to violence were included as predictors of item scores, traumatic life events did not emerge as a significant predictor of item scores across all analyses. This seems to suggest that, in the present sample, trauma was not related to Prime item scores in either race group when accounting for the effect of exposure to community violence and experiences of discrimination. Although there is a strong link in the literature between trauma and psychosis [35,60,61], it may be the case that factors such as community violence or discrimination account for some of this effect in our sample, or that such factors may share common pathways to produce the identified link between trauma and psychosis. For example, Stowkowy and colleagues (2016) evaluated the predictive power of trauma, bullying, and perceived discrimination in predicting conversion to psychosis among individuals at a clinical high-risk for psychosis [62]. The authors reported that when including all three factors in the model, only discrimination significantly predicted later conversion to psychosis. Similarly, in a series of studies examining traumatic life experiences, discrimination, and dissociation in racial and ethnic minority youth, Polanco-Roman and colleagues (2016) [63] found that racial discrimination was associated with dissociative symptoms. In another study by Anglin and colleagues (2015) [64], the relation between traumatic life events and attenuated positive psychotic symptoms was mediated by dissociation, particularly for individuals of self-identified Black race. Such work may point to the impactful role discrimination may play in the relation between trauma and psychosis.

### 4.4. Limitations and Future Directions

Given the exploratory nature of our analyses, the present findings should be interpreted with caution. Although this is primarily a non-help seeking sample, participants were not excluded for current mental health treatment. Nonetheless, as the majority of participants were not receiving services (72%), findings may not generalize to clinical samples. Additionally, the sample primarily identified as female gender (78%); although men report higher rates of trauma exposure, women are diagnosed with PTSD at higher rates [65]. Thus, the current findings may reflect sampling bias. Replication is needed in independent samples with greater sample sizes to establish whether the current findings represent true relations or are a reflection of sampling variability. Similarly, the complex nature of the relation between trauma, discrimination, community violence, and psychosis experiences prohibits an interpretation of the exact nature of how these factors may be impacting the validity of Prime Screen items. More work, both cross-sectional and longitudinal, is vital to elucidate whether biases exist in measures of psychosis experiences like the Prime Screen and, if so, to understand the exact nature of these biases. In the same vein, more work exploring the impact of other relevant factors including religion, spirituality, and ethnic identity, among others, on Prime Screen psychometrics is needed.

These limitations and concerns notwithstanding, the current study builds on the body of work evaluating the psychometric performance of measures of psychosis experiences. We found that acceptability of the overall component structure of the Prime Screen, as well as specific items, seems to be associated with race, discrimination, and community violence. These results highlight potential deficits in the psychometric validity of a commonly used measure of psychosis experiences and suggest that use of the Prime Screen across diverse groups may require further psychometric evaluation, or even the modification or removal of some items. Such efforts may aid in further clarifying the complex relations between psychosis experiences and factors such as trauma, discrimination, and community violence among diverse groups of individuals.

## Figures and Tables

**Table 1:** Descriptive Statistics and Frequencies for Study Variables.

	Total Sample	Black Participants	White Participants
	Frequency (%)/Mean (SD)		
**Gender**			
Female	191 (78%)	64 (78%)	127 (78%)
Male	52 (21%)	18 (22%)	34 (21%)
Non-binary/third gender	1 (<1%)	0 (0%)	1 (1%)
**Prime Screen Risk Status**			
Low-risk	149 (61%)	46 (56%)	103 (64%)
At-risk	95 (39%)	36 (44%)	59 (36%)
**Experiences of Discrimination**			
Total Score	0.95 (1.55)	1.81 (1.90)	0.54 (1.15)
0 domains of discrimination	127 (52%)	22 (27%)	105 (65%)
1–3 domains of discrimination	69 (28%)	34 (17%)	35 (21%)
4–6 domains of discrimination	15 (6%)	11 (13%)	4 (2%)
7+ domains of discrimination	3 (1%)	2 (2%)	1 (1%)
Missing/Prefer not to respond	30 (12%)	13 (16%)	17 (11%)
**Community Exposure to Violence**	1.45 (1.45)	1.39 (0.40)	1.48 (0.43)
**Age**	21.99 (5.07)	21.11 (3.05)	22.44 (5.79)
**Prime Screen Total Score**	16.45 (14.07)	16.65 (14.47)	16.36 (13.91)
**Life Events—Self Total Score**	2.34 (1.97)	2.12 (1.94)	2.45 (1.99)

**Table 2:**
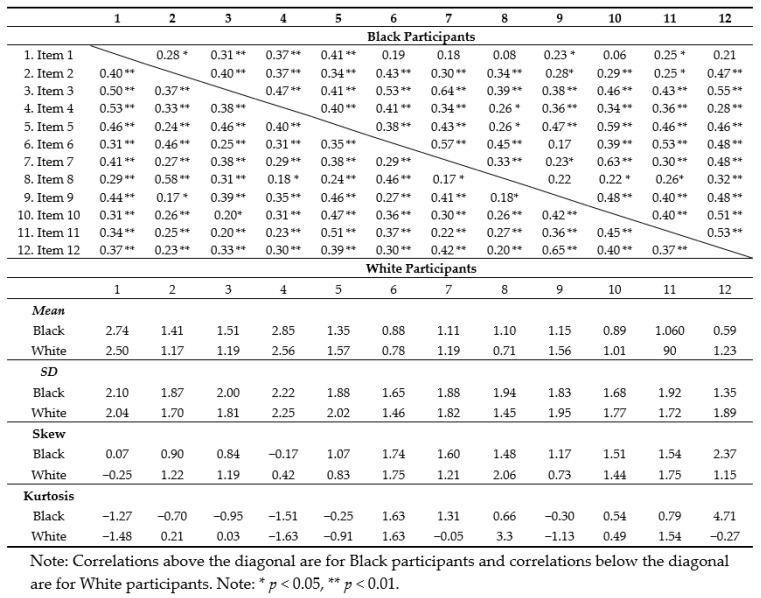
Correlation coefficients, descriptive statistics, and normality estimates for Prime Screen items.

**Table 3:** Component Loadings for Principal Components Analysis of Prime Screen Items.

	Black/African American		White/Caucasian	
	1	2	1	2
**1. I think that I have felt that there are odd or unusual things going on that I can’t explain**	**0.54**	−0.12	**0.72**	0.14
*2. I think that I might be able to predict the future*	*0.46*	*0.49*	*0.47*	*0.72*
**3. I may have felt that there could possibly be something interrupting or controlling my thoughts, feelings, or actions**	**0.72**	0.35	**0.63**	0.26
**4. I have had the experience of doing something differently because of my superstitions**	**0.65**	0.00	**0.62**	0.18
**5. I think that I may get confused at times whether something I experience or perceive may be real or may just be part of my imagination or dreams**	**0.78**	−0.05	**0.75**	0.02
*6. I have thought that it might be possible that other people can read my mind, or that I can read other’s minds*	*0.59*	*0.58*	*0.57*	*0.45*
7. *I wonder if people may be planning to hurt me or even may be about to hurt me*	*0.60*	*0.49*	**0.65**	−0.01
*8. I believe that I have special natural or supernatural gifts beyond my talents and natural strengths*	*0.37*	*0.64*	*0.43*	*0.69*
9. I think I might feel like my mind is “playing tricks” on me	**0.72**	−0.24	**0.77**	−0.29
**10. I have had the experience of hearing faint or clear sounds of people or a person mumbling or talking when there is no one near me**	**0.76**	0.04	**0.66**	0.01
**11. I think that I may hear my own thoughts being said out loud**	**0.73**	0.06	**0.65**	0.06
**12. I have been concerned that I might be “going crazy”**	**0.68**	0.29	**0.75**	−0.24
% of variance	41.63%	12.54%	41.67%	12.19%

Note: All Prime Screen items were normalized via a natural log transformation before principal components analyses were estimated. Note: Items with high loadings (≥ 0.5) on one component and non-significant loadings (≤ 0.32) on another are formatted in bold above. Those with significant cross-loadings across both components or without strong loadings on either component are formatted in italics and underlined above. Note: The percentage of variance accounted for by each component above is post-rotation.

**Table 4:** Correlation coefficients for Prime Screen items and Mental Well-Being Scores.

	Prime Items											
	1	*2*	3	4	5	*6*	*7*	*8*	9	10	11	12
Mental Well-Being												
Black	−0.17	*−0.01*	−0.20	−0.06	−0.13	*−0.04*	*−0.08*	*0.01*	−0.15	−0.17	−0.10	−0.23 *
White	−0.22 **	*−0.04*	−0.19 *	−0.17 *	−0.22 **	*−0.01*	−0.24 **	*0.09*	−0.33 **	−0.10	−0.09	−0.32 **

Note: * *p* < 0.05, ** *p* < 0.01. Note: Component 2 items (“poor fit”) are italicized above.

**Table 5:** Multivariate Linear Regression Models Predicting Good Fit Prime Screen Item Scores.

**Black/African American**				
**Prime Items**	**Traumatic Life Events**	**Experiences of Discrimination**	**Community Violence**	***R*^2^**
Item 1	0.08	−0.01	0.00	0.04
Item 3	−0.01	−0.10	0.33	0.07
Item 4	0.01	0.03	0.40	0.06
Item 5	−0.04	−0.03	0.19	0.02
Item 9	0.03	−0.04	0.19	0.02
Item 10	0.00	−0.08	0.39	0.07
Item 11	0.09	−0.08	0.10	0.06
Item 12	0.02	−0.04	0.09	0.02
**White/Caucasian**				
**Prime Items**	**Traumatic Life Events**	**Experiences of Discrimination**	**Community Violence**	***R*^2^**
Item 1	0.01	0.02	0.51 **	0.08
Item 3	0.02	0.08	0.13	0.04
Item 4	0.03	−0.04	0.73 ***	0.15
Item 5	−0.03	0.02	**0.35 ^†^**	0.03
Item 9	0.02	−0.02	0.74 ***	0.16
Item 10	0.00	−0.08	0.62 ***	0.13
Item 11	0.02	0.01	**0.26 ^†^**	0.04
Item 12	0.03	−0.03	0.51 **	0.10

^†^*p* < 0.10; ** *p* < 0.01; *** *p* < 0.001.

**Table 6:** Multivariate Linear Regression Models predicting Poor Fit Prime Screen Item Scores.

**Black/African American**				
**Prime Items**	**Traumatic Life Events**	**Experiences of Discrimination**	**Community Violence**	***R*^2^**
Item 2	−0.00	0.12 *	−0.07	0.10
Item 6	0.01	−0.07	0.06	0.04
Item 7	0.01	−0.10 *	0.39	0.10
Item 8	−0.00	−0.04	0.28	0.03
**White/Caucasian**				
**Prime Items**	**Traumatic Life Events**	**Experiences of Discrimination**	**Community Violence**	***R*^2^**
Item 2	0.02	0.02	**0.28 ^†^**	0.04
Item 6	−0.01	−0.10 *	0.32 *	0.06
Item 7	0.04	−0.11 *	0.38 *	0.09
Item 8	0.03	0.09 *	−0.05	0.05

^†^*p* < 0.10; * *p* < 0.05.
